# Aspect-ratio driven evolution of high-order resonant modes and near-field distributions in localized surface phonon polariton nanostructures

**DOI:** 10.1038/srep32959

**Published:** 2016-09-13

**Authors:** Chase T. Ellis, Joseph G. Tischler, Orest J. Glembocki, Francisco J. Bezares, Alexander J. Giles, Richard Kasica, Loretta Shirey, Jeffrey C. Owrutsky, Dmitry N. Chigrin, Joshua D. Caldwell

**Affiliations:** 1 U.S. Naval Research Laboratory, Washington, DC, USA; 2 American Society for Engineering Education, Washington, DC, USA; 3 Center for Nanoscale Science and Technology, National Institutes of Standards and Technology, Gaithersburg, MD, USA; 4 I. Institute of Physics (IA), RWTH Aachen University, Aachen, Germany

## Abstract

Polar dielectrics have garnered much attention as an alternative to plasmonic metals in the mid- to long-wave infrared spectral regime due to their low optical losses. As such, nanoscale resonators composed of these materials demonstrate figures of merit beyond those achievable in plasmonic equivalents. However, until now, only low-order, phonon-mediated, localized polariton resonances, known as surface phonon polaritons (SPhPs), have been observed in polar dielectric optical resonators. In the present work, we investigate the excitation of 16 distinct high-order, multipolar, localized surface phonon polariton resonances that are optically excited in rectangular pillars etched into a semi-insulating silicon carbide substrate. By elongating a single pillar axis we are able to significantly modify the far- and near-field properties of localized SPhP resonances, opening the door to realizing narrow-band infrared sources with tailored radiation patterns. Such control of the near-field behavior of resonances can also impact surface enhanced infrared optical sensing, which is mediated by polarization selection rules, as well as the morphology and strength of resonator hot spots. Furthermore, through the careful choice of polar dielectric material, these results can also serve as the guiding principles for the generalized design of optical devices that operate from the mid- to far-infrared.

The quasiparticle excitation that arises from interactions between coherent charge oscillations and electromagnetic radiation, known as an optical polariton, gives rise to a wide variety of near-field optical phenomena that enable the control and manipulation of light in the subdiffractional regime^1^,^2^. This regime is not ordinarily accessible via free-space optics, which is limited by the Abbe diffraction limit. However, under the right conditions, light impinging upon a nanostructure much smaller than the wavelength of light can excite optical polariton resonances that strongly confine fields near the surface, resulting in the enhancement of local electromagnetic fields and emission/absorption rates of nearby devices. In such situations these resonances are able to greatly alter the near-field radiation pattern at the nanoscale, which has had a profound influence on nanophotonic technologies, such as waveguides^3^,^4^,^5^, light sources^6^, near-field optics^7^,^8^,^9^, solar cells^10^,^11^, chemical sensors^12^, biosensors^13^,^14^, photonic circuitry^15^,^16^,^17^, and even cancer therapy^18^. Thus far, the majority of these technologies exploit electron plasmas in metals in order to excite and harness the power of optical polaritons (surface plasmon polaritons, SPPs) in the ultraviolet to near-infrared spectral regimes. Due to the high dispersion in the real part of the dielectric function and the corresponding steady increase in optical losses in the mid- to far-infrared regimes, the performance of metals for infrared nanophotonic applications is limited^19^. An alternative system that is not subject to these limitations employs polar dielectric crystals, which are capable of supporting phonon-mediated, collective oscillations of bound lattice charges that sustain a plasmonic-like, subdiffractional excitation over the infrared spectral range bounded by the longitudinal (LO) and transverse (TO) optic phonon frequencies (*ω*_*LO*_ and *ω*_*TO*_), known as the Reststrahlen band^20^,^21^,^22^,^23^. Such excitations, known as surface phonon polaritons (SPhPs), usually occur in the mid- to long-wave infrared spectral regime and are subject to significantly lower losses, in comparison to metals, in the infrared due to the long lifetimes of optic phonons (*τ* ≈ 1–100 ps) from which they are derived. These lifetimes are over an order of magnitude longer than the scattering times of free carriers in metals^24^. Moreover, low optical losses are of distinct importance for potential applications, as evidenced by the higher figures of merit reported, such as the quality factor^21^,^25^,^26^ and Purcell factor^21^, for SPhP materials. As such, these qualities provide an opportunity for SPhP materials to greatly influence nanophotonic technologies in the infrared spectral regime^27^,^28^. However, the realization of such opportunities hinges on the ability to understand, control, and manipulate the center wavelength, polarization, and near-field radiation patterns of the plasmonic-like resonances that are excited in polar dielectric materials, which is the main concern of this article.

For isolated nanoparticles with dimensions much smaller than the wavelength of light (*d*≪*λ*), the incident electric field is nearly homogeneous over the space occupied by the particle (quasi-static regime). In this regime, considering the case of a non-interacting nanosphere, the dominant localized optical polariton resonance consists of a simple dipole that is aligned parallel to the incoming light polarization. However, altering the nanoparticle environment, shape, and size can significantly modify this resonance, leading to new resonances that deviate from such simplified conditions. For example, by increasing the nanostructure size, retardation effects within the optical resonator become important, that is, the phase of the incident electric field varies over the nanostructure dimensions, resulting in the excitation of higher order multipoles^29^,^30^,^31^. In addition, symmetry breaking, which is introduced by interactions between adjacent nanostructures^32^ or a nanostructure and a nearby substrate^33^,^34^, can result in the hybridization of allowed dipole and unallowed higher-order resonant modes. Furthermore, changing the shape and therefore the symmetry of plasmonic nanoparticles has a profound effect on the various localized optical polariton resonances that can be optically excited. This effect is clearly seen by comparing the extinction spectra for isolated spherical and cubic nanoparticles of subwavelength dimensions, where the quasi-static regime (*d*≪*λ*) is applicable and retardation effects are negligible^35^,^36^. While the nanosphere spectra consists of only a single dipole resonance, the isolated nanocube spectra reveals many resonances that include this dipole resonance, as well as many other higher-order modes with more complicated near-field radiation patterns^36^,^37^,^38^,^39^. The simplicity of the spherical optical resonance stems from the fact that this geometry is only capable of supporting a homogeneous polarization in the quasi-static regime. However, for the case of the cube, corners and edges are able to concentrate charge, leading to many non-homogeneous polarization configurations^39^. By changing and controlling the configuration of charge concentration, one also controls the areas where near-field enhancement occurs. To date, optical studies of localized SPhP resonances have mainly focused on nanoresonators with cylindrical symmetry, where the excitation source is mainly oriented perpendicular to the symmetry axis. With such geometries, light is able to efficiently couple to dipole and monopole resonances^21^,^40^,^41^. However, thus far, the excitation of higher-order localized SPhP resonances with more exotic near-field radiation patterns, such as those theoretically predicted and observed for cubic plasmonic nanoparticles^37^,^38^,^39^, have remained absent in the literature.

In this article, we experimentally and theoretically explore the far- and near-field behavior and aspect-ratio (shape) driven evolution of both transverse and longitudinal, high-order localized SPhP resonances that occur in arrays of three-dimensional rectangular pillars. These structures are etched into semi-insulating, 4H silicon carbide substrates, similar to efforts previously reported from our group^21^,^41^. A combination of the high structural asymmetry of these optical resonators, low optical losses, as well as the relative size of each pillar axis, leads to the excitation and spectroscopic observation of many high-order resonances with complex, three-dimensional, near-field radiation patterns. Through polarized reflectance measurements and finite-element-method simulations, we are able to explore the polarization selection rules of the localized SPhP resonances and their evolution with increasing aspect ratio (*AR*). Interestingly, for many of the resonances we find that not only does the excitation energy vary with changing aspect ratio, but that the modal order also evolves as this quantity is increased, thus resulting in a splitting of the aspect ratio driven dispersion of specific resonances. This is in contrast to resonances in metallic nanostructures that exhibit plasmonic resonances with an evolving excitation energy, while preserving the modal order. Furthermore, despite the degree of control over both the resonance energies and near-field profiles that varying *AR* can provide in three-dimensional rectangular structures, such an investigation has remained elusive in literature, even for metal-based SPP systems. This is largely due to the difficulty in producing three-dimensional, rectangular plasmonic nanoparticles with well-controlled dimensions, a high degree of monodispersity, and low optical losses, where the latter two properties significantly broaden any resonances, resulting in strongly overlapping modes that are difficult to separate and/or distinguish. In this work, such challenges are overcome by the low optical losses of SPhPs in silicon carbide and the use of top-down fabrication techniques that produce highly uniform pillar arrays^21^, providing exceptionally narrow localized SPhP resonances. The understanding we present here is critical to both sensing and enhanced emitter applications, where knowledge of the polarization selection rules enables one to tailor resonances in order to achieve more efficient near-field coupling between the nanostructure and the local emitter, molecule or other polariton source or for the more efficient design of polariton-based thermal emitters, with the modal behavior dictated by the near-field radiation patterns^42^,^43^.

To study the excitation of multipolar localized SPhP resonances, we perform polarized reflectance measurements on fixed-height (*H* = 950 nm), fixed-width (*W* = 400 nm), rectangular-shaped, semi-insulating 4H-SiC pillars that vary in length (*L* = 400–4800 nm) and therefore aspect ratio (*AR* = *L*/*W* = 1–12). These pillars are patterned on a square grid with a pitch (*P*) that is 500 nm larger than the pillar length (i.e., *P* = *L* + 500 nm) to reduce coupling between neighboring pillars while maintaining a high filling fraction. Figure 1a,b show oblique and top-view scanning electron microscope (SEM) images of a representative SiC pillar array that depicts the general geometry for the samples studied. To excite localized SPhP resonances, polarized reflectance measurements are performed with the incident polarization oriented either parallel or perpendicular to the long axis of the pillars, as indicated in Fig. 1. By selecting a single polarization orientation for each measurement, we are able to effectively isolate localized SPhP resonances that are excited along the length and width of the pillar, since, in general, the incoming light couples to resonant modes that have a net dipole moment component that is oriented parallel to the incoming polarization.

## Results and Discussion

**Figure 2:**
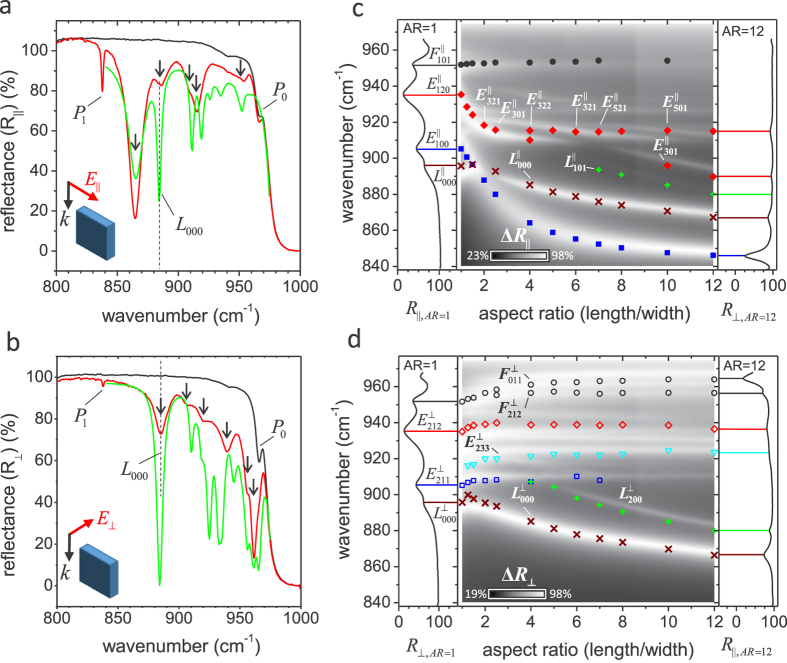
Measured and simulated reflectance of SiC pillar arrays. (**a,b**) Results of reflectance measurements performed on unpatterned SiC (black curve) and the *AR* = 4 pillar array (red curve), as well as the simulated reflectance (green curve). All measurements and simulations are performed with a 22° off-normal angle of incidence and incoming polarization oriented (**a**) parallel (*R*_||_) and (**b**) perpendicular (*R*_⊥_) to the elongated edge of the pillars (*L*). SPhP bands are indicated by arrows and zone folded LO bands are labeled *P*_0_ and *P*_1_. (**c,d**) Measurements and simulations of the aspect ratio evolution of localized SPhP resonance energies for the (**c**) parallel and (**d**) perpendicular polarizations. The middle panel for (**c,d**) demonstrates how the measured spectral position of the localized SPhP resonances evolve with pillar aspect ratio (symbols), for their respective incident polarizations. The measured spectral positions of localized SPhP resonances are overlaid for comparison on the finite element method simulations of the differential reflectance 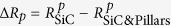, which is represented by the density plot, with white and black shaded areas corresponding to low and high reflectance, respectively. The left and right panels panels (**c,d**) show the substrate normalized reflectance measurements of the *AR* = 1 and *AR* = 12 pillar arrays, respectively.

The influence of the pillars upon the resonant spectra is clearly observed in Fig. 2a,b, which show the results of polarized FTIR reflectance measurements performed on un-patterned 4H-SiC (black curve) and the *AR* = 4 pillar array (red curve), with a 22° weighted average angle of incidence and polarization nominally oriented parallel (*R*_||_) and perpendicular (*R*_⊥_) to the length of the pillars, respectively. Due to the conical illumination geometry, both s and p polarized light are simultaneously present for *R*_||_ and *R*_⊥_ measurements (see Figure S1 in Supporting Information). As demonstrated in the black spectrum in Fig. 2a,b, between *ω*_*TO*_ = 797cm^−1^ and *ω*_*LO*_ = 973 cm^−1^ the bulk SiC exhibits nearly 100% reflectance. This is due to the effective screening of radiation by oscillating lattice charges driven by the polar optical phonons. Comparison of the pillar reflectance spectra with that obtained for the bare substrate reveals clear deviations resulting from the excitation of localized SPhP resonances. As indicated by the arrows in Fig. 2a, the parallel polarization reveals five modes that are observed in the range of 864–953 cm^−1^. Similarly, for the perpendicular polarization, (Fig. 2b), six resonances appear in the reflectance spectrum, over the spectral range of 885–961 cm^−1^. For both polarizations, the sharp peaks that occur at 837 cm^−1^ (labeled as *P*_1_ in Fig. 2a,b) and 964 cm^−1^ (labeled as *P*_0_) are associated with zone-folded LO phonons of 4H-SiC with a reduced wave vector of *x* = 1 and *x* = 0, respectively^44^. Interestingly, the *P*_1_ mode is not infrared active, as confirmed by its absence from reflectance measurements of the unpatterned SiC substrate. However, the *P*_1_ phonon peak is observed with increasing amplitude as the localized SPhP resonances spectrally approach the *P*_1_ phonon frequency. The existence of coupling effects between the *P*_1_ phonon and localized SPhP resonances is further corroborated by the *AR* dependence of reflectance measurements that are shown in Figure S2 of the Supporting Information, where the *P*_1_ mode clearly strengthens as the localized SPhP resonances spectrally approach and cross through this phonon mode, resulting in a Fano-like interference during the crossing. This observation is consistent with our previous findings within cylindrical nanopillars^21^. Although interesting, a detailed discussion of this phenomena is beyond the scope of this paper, therefore we defer further studies of this interaction to a later date.

As demonstrated in Fig. 2a,b, for elongated pillars with structural asymmetry (i.e., *L* ≠ *W* ≠ *H*), each polarization orientation gives rise to distinct localized SPhP resonances that are known to be associated with excitations along the length, width (transverse resonances) and height (longitudinal resonances) of the pillars. This is similar to plasmonic nanostructures with structural asymmetries such as triaxial ellipsoids^24^,^45^,^46^,^47^. For the *AR* = 4 reflectance measurements, only a single localized SPhP resonance (labeled as *L*_000_ and marked by the vertical dashed line in Fig. 2a,b) deviates from this behavior and is observed for both orthogonal polarizations at the same energy. Previously, it was demonstrated that this mode has a longitudinal nature and was referred to as a ‘monopolar’ resonance due to the abnormal near-field distribution that results from the attachment of the pillars to the negative permittivity SiC substrate^21^,^41^.

To model the far- and near-field resonant behavior of the rectangular pillars, we perform full-wave electromagnetic simulations of the polarized pillar reflectance using the finite element method (FEM) calculations in COMSOL Multiphysics. The pillar geometry is modeled using the nominal values for the pillar array *L*, *W*, *H* and *P*, and includes effects due to the rounding of the pillar corners and edges, to ensure that the geometry matched the fabricated pillar structures as closely as possible (see Methods). Simulations of *R*_||_ and *R*_⊥_ for the *AR* = 4 pillar array (green curve in Fig. 2a,b, respectively) semi-quantitatively agree with the corresponding reflectance measurement. Overall, despite differences in the linewidths, presumably due to imperfect fabrication, the simulated spectral positions of the resonances are in excellent agreement with measurements. In addition, we also simulate the polarized reflectance spectra (density plot) for *AR* = 1–12, as shown in Fig. 2c,d for parallel and perpendicular polarization, respectively. These plots present the differential reflectance (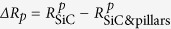, where *p* = || or ⊥ denotes the polarization orientation). For both polarizations, trends in the simulated resonance shifts displayed in the density plots (white shaded, low-reflectance regions) are in excellent agreement with the experimentally measured resonance positions (symbols) for all *AR* studied. Two additional resonances do appear in the simulations of the parallel polarization that are absent from reflectance measurements (found at spectral positions *ω* = 926 and 935 cm^−1^ for *AR* = 4). However, due to the significantly weaker amplitudes of these resonances in comparison to the other measured modes, it is possible that these resonant features are below the detection limit of our system or are difficult to detect due to additional broadening.

**Figure 3:**
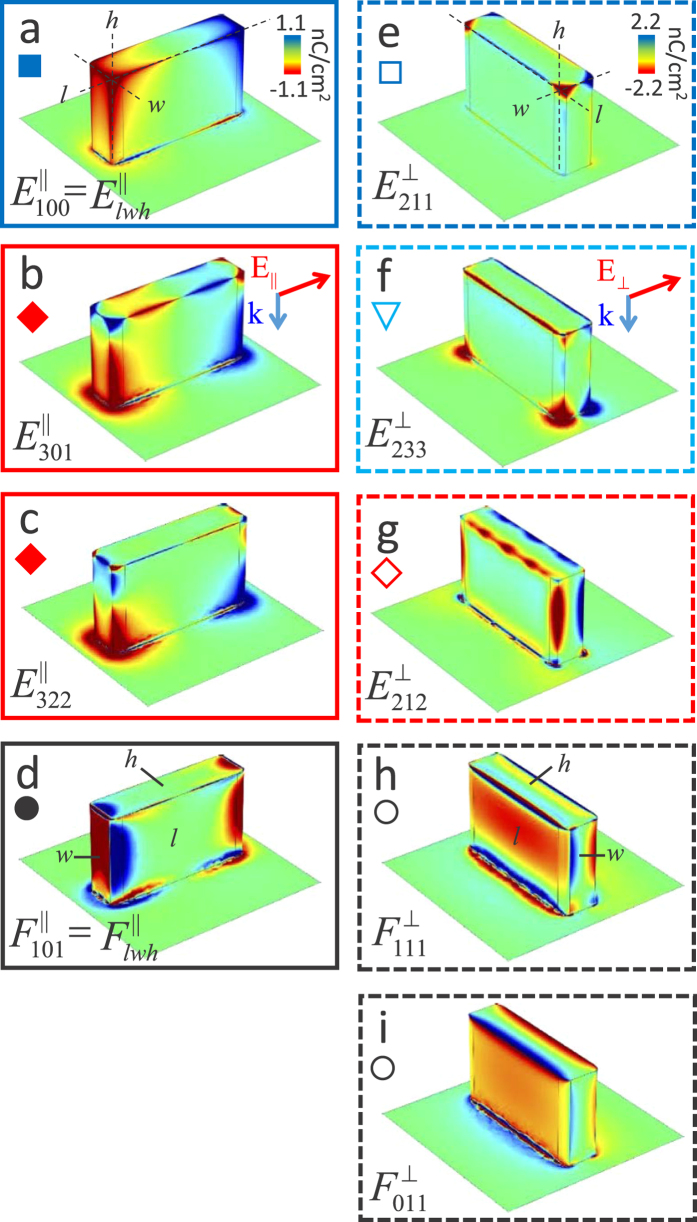
Near-field behavior of localized surface phonon polariton resonances for *AR* = 4 pillars. Surface charge densities for transverse localized SPhP resonances excited by light polarized (**a–d**) parallel and (**e–i**) perpendicular to the pillar length and normally incident radiation (see Methods section for calculation details). Red and blue shading represent charge densities with opposite polarity. Panels (**a–i**) are plotted using separate color scales (see panels a and e insets). The parameters *l*, *w* and *h* that enumerate the number of charge nodes are determined by the edges and faces indicated by their respective guidelines in panels (**a,d,e,h**). In addition, the symbols shown for each panel corresponds to the symbols used for each similarly marked resonance shown in the spectra of Fig. 2c,d.

In addition to simulations of the pillar reflectance spectra, we are also able to extract the near-field charge distributions for each localized SPhP resonance. Figure 3 shows the simulated transverse near-field charge distributions for the *AR* = 4 pillar array at normal incidence. Although we have also calculated the charge distributions for an angle of incidence of *θ* = 22° to match our experimental conditions, we find that the pillar charge profiles are difficult to interpret due to charge inhomogeneities that are induced by non-uniform illumination of the pillars from a unidirectional light source; therefore, we limit our discussion to the case of normal incidence. We do note that as shown in Figure S3 of the Supporting Information, simulations reveal negligible changes in the spectral positions of transverse localized SPhP resonances when excited by normal or off-normal incident light, indicating that the fundamental behavior of the resonances is independent of incident direction (as discussed later this is not the case for longitudinal resonances). As shown in Fig. 3, the resonant charge density profiles of the *AR* = 4 pillars take on two distinct forms; either the charge is primarily distributed along the 1) pillar edges and corners or 2) pillar faces. Based on this, hereafter we will refer to these distributions as edge and face modes, respectively. These findings are consistent with both calculations^39^ and measurements^38^ of the modal charge distributions for the strongest resonances of cube-shaped nanoresonators. However, we find that the rectangular nanopillars studied in this work are capable of supporting many higher order modes that can be thought of as multipolar extensions of the modes found in nanocubes. In Figs 2 and 3 we label transverse resonances as 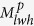, where *M* = *E* or *F* to denote the resonance as an edge or face mode, respectively; *p* = ⊥ or || identifies the excitation polarization (as defined earlier) and *l*, *w* and *h* are integers that indicate the number of charge nodes (i.e., points where charge polarity changes sign) that exist along the pillar edges or faces along the length, width, and height of the pillars, respectively. For edge modes, the indices *l* and *w* are determined by the number of nodes along the top edges of the pillar where the charge density profile is least affected by substrate effects and the most defining characteristics of the modes occur. Longitudinal resonances are similarly labeled as 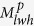; however, for these resonances we do not differentiate between face and edge modes, rather we use *M* = *L* to denote the longitudinal nature of these modes.

For the parallel polarized case, the lowest energy resonance 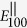 is identified as a transverse dipole mode (see Fig. 3a), as indicated by the existence of only a single node along the pillar length. This mode has been well studied in cylindrical SiC pillar arrays^21^,^41^. In contrast to the parallel polarization, at *AR* = 4, the lowest energy transverse resonance 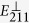 that is excited by the perpendicular polarization is not a simple dipole mode, as shown in Fig. 3e. Rather, this mode exhibits a modal profile with charge separation along all three pillar axes, including the pillar length that is orthogonal to the incident polarization, demonstrating the fact that rectangular pillars are able to support non-homogeneous polarization configurations, not accessible in pillar geometries with higher symmetry (e.g., cylinders). As shown in Fig. 3b–i, higher energy transverse resonances correspond to higher-order localized SPhP excitations that exhibit many charge density nodes along the three pillar axes. In general, the excited modes are either characterized by high charge concentrations at the pillar corners and edges (edge modes, 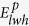) or charge distributed across the pillar faces (face modes, 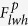). Similar to the results of previous calculations for ionic crystal cubes^39^ and experiments on metallic nanocubes^37^,^38^, we find that the majority of resonances are edge modes that are excited at photon energies lower than those needed to excite face modes. For both types, the presence of the substrate strongly modifies the charge distribution near the base of the pillar, which breaks the top-bottom charge distribution symmetry that is typical of isolated cube shaped plasmonic nanoparticles^37^,^39^. This is especially apparent for 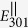 and 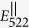 (see Fig. 3b,c, respectively), which exhibit many charge density nodes along the top of the pillar, but only show a single node along the pillar base. This top-bottom asymmetry of the charge distribution leads to a small longitudinal polarization component for the resonances. However, by calculating the dipole moments of the resonant charge distributions along the pillar axes, we find that this longitudinal component of the mode is over an order of magnitude weaker than the dominant transverse component.

As briefly introduced in Fig. 2c,d, simulations and measurements show that resonances can be tuned over a broad spectral range by elongating only a single axis of the rectangular pillars (*AR* = 1–12). Resonances can be further tuned by manipulating the dimensions of all three pillar axes, as done for ellipsoidal plasmonic nanoparticles^45^; however, to simplify our discussion we concentrate on varying only a single pillar axis. At *AR* = 1, both *R*_||_ and *R*_⊥_ yield equivalent resonances due to the square symmetry of the pillar cross-sections. As *AR* is increased and cross-sectional symmetry is broken (i.e., *L* ≠ *W*), the 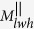 and 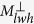 resonances spectrally separate, as observed by comparing the *AR* evolution of *R*_||_ and *R*_⊥_ modes shown in Fig. 2c,d (symbols), respectively. Thus, as previously discussed for the case of *AR* = 4 and further corroborated here, the rectangular cross-section results in the excitation of distinct resonances associated with each pillar axis. Furthermore, we find that as *AR* increases, the SiC pillars are capable of supporting additional higher order transverse modes that are not supported at smaller *ARs* (e.g., this is clear for the modes represented by ♦ symbols in Fig. 2c). In general, the transverse resonances excited by light polarized parallel (perpendicular) to the pillar length redshift (blueshift) with increasing *AR*. While care has been taken to reduce the interpillar coupling by separating pillars with a 500 nm gap, not all effects have been eliminated. Pitch dependent simulations (at fixed *AR*) indicate that most resonances exhibit a spectral redshift of less than 1 cm^−1^ due to coupling between neighboring pillars. However, simulations also show that 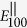 deviates from this behavior, revealing a spectral redshift of the resonance with respect to the 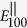 mode calculated for an uncoupled pillar array. As discussed in the Supporting Information (Figure S4), the redshift of 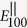 due to the coupling of pillars increases with aspect ratio.

**Figure 4:**
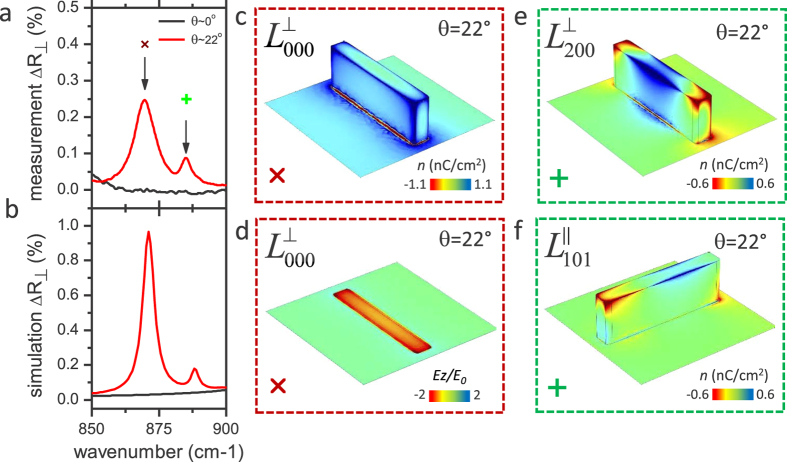
Comparison of resonances excited by normal and off-normal incident radiation. (**a**) Measured and (**b**) simulated reflectance spectra for the *AR* = 7 pillar array with off-normal (red curve) and near-normal (black curve) incident light. Symbols with arrows indicate the two longitudinal localized SPhP resonances that are quenched by extinguishing the polarization component oriented along the height of the pillar at normal incidence. (**c**) shows the surface charge density profile and (**d**) shows the electric field under the base of the pillar for the *L*_000_ monopole mode. (**e,f**) show the surface charge density profile for the parallel and perpendicular polarized modes for 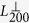 and 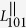, respectively. All modal profiles are shown for *AR* = 7 pillars. Symbols in the lower left corner of (**c–f**) are used to identify these resonances in Fig. 2c,d.

In contrast to the broad array of edge and face-oriented transverse modes, the so-called ‘monopole’ resonance, *L*_000_ remains spectrally degenerate for both polarizations over the entire range of *ARs*. In addition, we also find higher-order longitudinal resonances 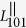 and 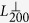 (+symbols shown in Fig. 2c,d, respectively) that are spectrally degenerate. These resonances occur at a higher frequency than *L*_000_ and are only optically active at larger aspect ratios (*AR* ≥ 4). Similar to *L*_000_, the longitudinal nature of 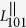 and 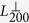 is confirmed by the spectral degeneracy of the resonances excited by both orthogonal polarizations and by angle of incidence measurements, revealing that a surface-normal polarization component is necessary in order to excite these modes. This is demonstrated in Fig. 4a, which shows the measured reflectance spectra of the *AR* = 7 nanostructure measured at *θ* ≈ 0° and 22° (see Methods section) with light polarized perpendicular to the pillar length. At off-normal angles (*θ* ≈ 22°) both *L*_000_ and 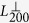 resonances are clearly observed (red curve); however, near normal incidence *θ* ≈ 0 (black curve) these modes are extinguished due to the absence of a longitudinal polarization component. This behavior is in excellent agreement with simulations, as shown in Fig. 4b. Although Fig. 4a,b only show the results of measurements and simulations performed with the perpendicular polarization, the parallel polarization measurements and simulations of *L*_000_ and 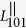 also exhibit this behavior. However, the demonstration of such behavior is much clearer for *R*_⊥_, since *L*_000_ and *L*_200_ are more clearly spectrally separated from neighboring resonances. Figure 4c shows the simulated charge density profile for *L*_000_ that occurs near *ω* = 884 cm^−1^ at *AR* = 7. A significant portion of charge is concentrated near the base of the pillar, which is compensated by charge of the opposite polarity that is distributed across the substrate surface between the pillars. This is consistent with a monopole mode that has been discussed in previous work^21^,^40^,^41^,^48^. The monopole nature of *L*_000_ is further corroborated by a strong concentration of the electric field underneath the pillar base, as indicated by the field plot shown in Fig. 4d. As predicted in prior work^21^,^41^, and as evidenced by our angle-dependent reflectance measurements, such a mode is not directly excited, rather it results from a longitudinal excitation that interacts with the substrate, yielding a monopolar charge distribution and near-field profile for both incident polarizations. Interestingly, simulations reveal distinct charge density profiles for the two spectrally degenerate resonances 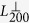 and 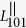, shown in Fig. 4e,f, respectively, despite the fact that these modes are spectrally degenerate. Furthermore, these resonances also differ by the fact that 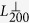 couples to radiation at all *AR* > 4 (+symbols in Fig. 2d), while 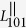 is only observed at *AR* > 7 (+symbols in Fig. 2c). These modal differences for the two polarizations are especially interesting given that the resonant energies of the two modes are nearly identical, with a separation of only Δ*ω* = 0.06 cm^−1^ according to simulations. This spectral separation is even smaller than the simulated spectral separation between the monopole mode excited by each polarization, Δ*ω* = 0.35 cm^−1^, where the modal profiles are nearly identical for both orthogonal polarizations (see Supporting Information Figure S5). In general, the *L*_000_, 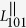 and 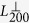 charge density distributions, shown in Fig. 4c,e,f, respectively, are preserved throughout the *AR* range where the modes are observed, making the near-field characteristics of these resonances highly predictable.

The combined data from reflectance measurements and simulations reveal three distinct forms of localized SPhP modes that have different *AR* and polarization dependencies: 1) longitudinal (discussed above) and 2) transverse resonances, both of which retain their near-field charge distributions with changing *AR* and 3) transverse resonances with modal profiles that evolve with *AR*. For the second type, the retention of the near-field distribution results in a ‘stretching’ of the modal profile without changing the overall number nodes as the pillars are elongated (i.e., 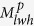 remains constant for all *ARs*). Due to this behavior we refer to these resonances as transverse ‘static’ modes. The observed modes that fall into this category include: 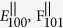 (resonances designated by ■ and ● symbols in Fig. 2c) as well as all of the transverse perpendicular modes 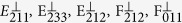 (resonances designated by symbols: □,▽,◊ and ○, respectively, in Fig. 2d). Similar to the longitudinally excited resonances, these transverse static modes are important due to the fact that their near-field modal profiles are highly predictable and remain unchanged regardless of the pillar *AR*. Furthermore, this behavior allows the near-field characteristics of these modes to be exploited over a wide range of energies, which is important for sensing applications that have specific frequency requirements and distinct spectral polarization selection rules.

**Figure 5:**
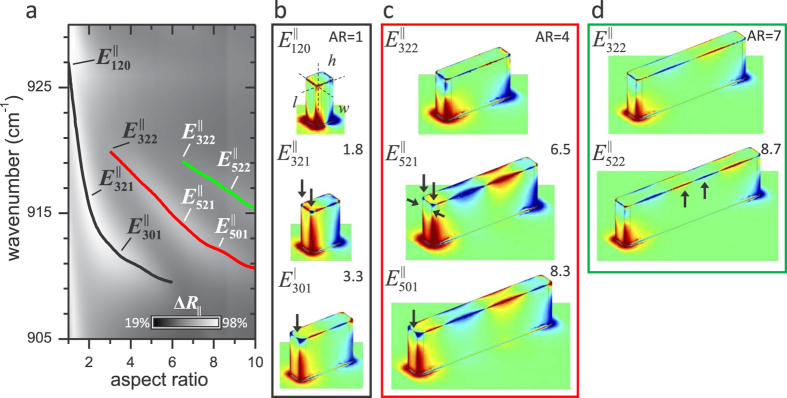
Evolution of surface charge distribution with *AR* for transverse dynamic resonances. (**a**) Simulated differential reflectance (Δ*R*_||_) of SiC Rectangular pillars with increasing *AR*, where white and black shaded areas correspond to high and low differential reflectance, respectively. The three curves indicate the three transverse dynamic resonances. (**b–d**) Shows the calculated redistribution of surface charge as *AR* increases for the three transverse dynamic resonances. Arrows mark major changes that occur in the charge distribution.

Similar to the transverse static modes, the third type of resonance is also transverse in nature. However, simulations indicate that the resonant charge density profiles of this third class are not static over the entire range of *ARs* investigated. Rather, as *AR* is increased, the charge is redistributed across the surface of the pillar in a non-trivial way, where the number of charge nodes are not preserved as the pillars are elongated (i.e., 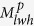 varies as *AR* changes). As such, we refer to these resonances as transverse ‘dynamic’. Such an effect is exemplified by the three resonances that appear in the simulations of Δ*R*_||_, shown in Fig. 5a (black, red, and green curves serve as guides to the resonance centers). Similar to all other parallel polarized resonances discussed previously, the simulated reflectance spectra show that these modes continuously redshift with increasing *AR*. However, as shown in Fig. 5b–d and indicated by the mode labels in Fig. 5a, for these three modes, charge is redistributed across the pillars as *AR* is increased. This is especially interesting, since the reflectance spectra show no indication of an evolving modal profile. This behavior is especially apparent for the resonance that is activated at the smallest *ARs* (black curve in Fig. 5a), where the charge density profiles for this mode exhibit three major charge distribution patterns (see Fig. 5b): 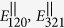 and 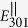, with modal transitions occurring near *AR* = 1.8 and 3.3. Similar behavior is observed for the other two resonances shown in Fig. 5c–d. This charge distribution evolution enables one to significantly alter the pillar charge distribution of resonances while having only a minor effect on the resonance energy. This property can have a significant impact on the local enhancement of nanoscale emitters and interaction with molecular vibrational modes for surface enhanced infrared absorption (SEIRA), since such modes enable one to custom tailor the near-field distributions. In addition, these effects also enable polarization and modal control of narrow-band, solid-state thermal emitters using localized SPhPs^42^,^43^.

## Conclusions

Through a combination of experiment and simulations we have demonstrated the ability to locally control the near-field profiles and resonant energies of localized SPhP resonances in rectangular pillars by adjusting the length of only a single pillar axis. The rectangular shape of the pillars plays a critical role in this control, since the squared off edges and corners of the pillar allows for the optical excitation of over 16 distinct, polarization-sensitive, localized SPhP modes that cannot be excited in structures with higher degrees of symmetry (e.g., spheres, cylinders, etc…). These optically-active resonances are extremely diverse in nature, ranging from a simple dipole mode to complex, high-order multipole resonances that exhibit intricate, three-dimensional, near-field profiles that are able to concentrate charge and electric fields along the faces, edges and/or corners of the pillars. As demonstrated in this work, the resonant energies for many of the observed localized SPhP resonances can be tuned without affecting the near-field profile of the mode by carefully selecting the length of pillar. This understanding provides a basis for exploiting the near-field characteristics of a particular mode over a wide energy range, which is especially important for applications where matching of the resonance energy and near-field distribution are required over a broader spectral range. In contrast, we also find that a series of resonances undergo a renormalization of the charge and electric field distribution with increasing *AR*, offering, in this case, flexibility in controlling the near-field characteristics of localized SPhP resonances, while retaining the resonance frequency. Overall, this understanding is imperative to achieving efficient coupling between the resulting modal symmetry of the pillars to that of a local emitter, molecule or other polariton source, where a matching of the near-field distribution can be critical. Furthermore, our demonstration that these resonances can be predicted using electromagnetic modeling, even for these highly complicated resonance spectra, clearly indicates that user-design of the resonance frequency, polarization properties and modal profile can be achieved.

## Methods

### Sample preparation

Silicon carbide pillars were formed on the surface of semi-insulating 4H-SiC substrates. To fabricate the pillars, electron-beam lithography and lift-off techniques were used to produce a Al/Cr hard mask pattern that defined the footprint of each pillar array. Subsequently, a reactive ion etch with equal partial pressures of SF_6_ and Ar at a power of 150 W was performed for 38 minutes in order to etch away the substrate, defining the height of the pillars. A wet chemical etch was used to remove the hard metal mask. To remove residual fluorine from the surface a commercial PlasmaSolv^®^ treatment was performed. Each of the resulting pillar arrays consist of rectangular pillars with a designed height of *H* = 950 nm, width of *W* = 400 nm, and a length of *L* = *AR* ⋅ *W*, where the length is dependent upon the aspect ratio (*AR*) selected for each array. The nominal aspect ratios of the pillars fabricated and measured are *AR* = 0.5, 0.75, 1, 1.25, 1.5, 1.75, 2, 2.5, 4, 5, 6, 7, 8, 10, 12. The pillars are designed on a square lattice with a pitch of *P* = *W* + 500 nm, which reduces coupling between neighboring pillars while maintaining a high filling fraction. In general, the length and width of the pillars is 16 ± 4% smaller than the nominal dimensions, while the pillar aspect ratios differ from the designed values by ±5%, as informed by SEM.

### Reflectance spectroscopy

Reflectance measurements are performed using a Bruker 80v spectrometer with microscope attachment (Hyperion 1000). The microscope is fitted with an all-reflective, reverse Cassegrain, microscope objective that illuminates the sample with a range of incident angles that varies over the range of *θ* = 15–30°. As depicted in Figure S1 of the Supporting Information, the microscope objective is not able to provide a single plane of incidence. Therefore, the conical illumination geometry results in the simultaneous measurement of both s and p polarized light, where the latter contains a polarization component oriented along the height of the pillar that is able to excite longitudinal resonances. Near normal incidence reflectance measurements are performed with a mid-infrared, ZnSe focusing objective (Innovation Photonics) that illuminates the sample with a range of incident angles that varies from *θ* = 0–4.5°. A mid-infrared, linear wire-grid polarizer that is placed in the microscope beam path enables the incident radiation to be nominally polarized either parallel or perpendicular to the long edge of the pillars. Due to the finite size of the pillar arrays (50 × 50 *μ*m), a set of apertures are used to limit the microscope field of view, so only spectra from the pillar array is collected. The utilization of the slits reduces the angle of incidence from the ZnSe objective even further. Measurements are normalized to the reflectance of a gold mirror and the reflectance of the 4H-SiC substrate.

### Modeling

Near- and far-field optical properties are calculated by determining the Full wave, 3D electrodynamic solutions to Maxwell’s equations via the RF package of the finite element method simulation software COMSOL. For these simulations, the pillar geometry was represented as a triaxial cuboid with rounded corners and edges. The dimensions used for the rounding of the pillar edges and corners was informed by SEM images of the fabricated pillars. In addition to being more true to the actual structure of the fabricated pillars, the geometric rounding also reduces calculation artifacts and instabilities that occur for structures with sharp corners. Numerical simulations are performed on a single pillar while employing periodic boundary conditions in order to account for interactions between neighboring nanopillars. The reflectance spectra and surface charge density distributions were calculated over the spectral range of the SiC Reststrahlen band for aspect ratios in the range of *AR* = 1–12 at increments of Δ*AR* = 0.25. All simulations were performed with two different angles of incidence, *θ* = 0° and 22°, which approximate the weighted average angle of incidence for the two microscope objectives used in the reflectance measurements. Furthermore, in order to excite both longitudinal and transverse resonances the incident radiation is p-polarized in order to guarantee a polarization component oriented along the height of the pillar for off-normal angles of incidence. To improve clarity of the surface charge distributions for the transverse modes in Fig. 3, the simulations shown are calculated with normal incidence. Both normal and off-normal incident radiation simulations show surface charge concentrated in the same positions of the pillar. However, unlike the normal incidence case, off-normal radiation produces a gradient in the magnitude of the charge density across the pillar. This charge density gradient depends on the orientation of the incoming wave vector used in the simulation. Since longitudinal modes are only excited by off-normal radiation, the surface charge density profiles shown for these modes (Fig. 4) are calculated with a 22° angle of incidence.

## Additional Information

**How to cite this article**: Ellis, C. T. *et al*. Aspect-ratio driven evolution of high-order resonant modes and near-field distributions in localized surface phonon polariton nanostructures. *Sci. Rep*. **6**, 32959; doi: 10.1038/srep32959 (2016).

## Supplementary Material

Supplementary Information

## Figures and Tables

**Figure 1:**
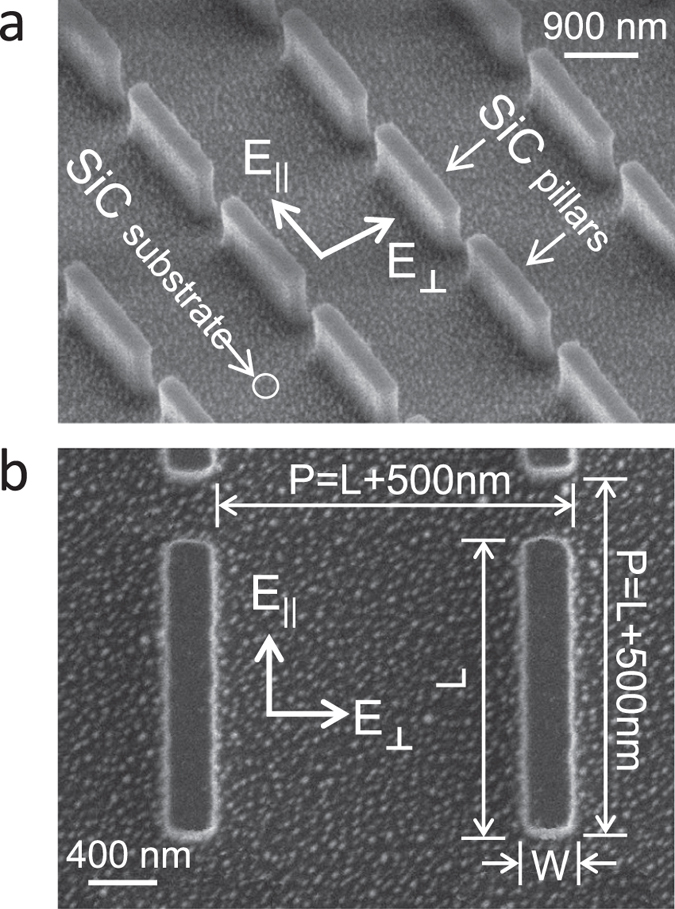
Scanning electron micrograph of SiC pillar array. (**a**) off-axis and (**b**) top view scanning electron micrograph of the AR = 6 optical resonator array. As indicated in both panels, the major component of the incoming light polarization is oriented either parallel or perpendicular to the elongated edge of the pillars. Panel (**b**) displays the important dimensions of the pillar array geometry.
