# Supplementary material for: Aspect-ratio driven evolution of high-order resonant modes and near-field distributions in localized surface phonon polariton nanostructures
Source: Sci Rep. 2016 Sep 13;6:32959. doi: 10.1038/srep32959 (PMC5020686; doi:10.1038/srep32959)
Supplement: Supplementary Information [file srep32959-s1.pdf]

Supporting information for “Aspect-ratio driven evolution of high-order resonant modes and near-field distributions in localized surface phonon polariton nanostructures”

Chase T. Ellis[1], Joseph G. Tischler[1], Orest J. Glembocki[1], Francisco J. Bezares[1,2], Alexander J. Giles[1], Richard Kasica[3], Loretta Shirey[1], Jeffrey C. Owrutsky[1], Dmitry N. Chigrin[4] and Joshua D. Caldwell[1].*

1. U.S. Naval Research Laboratory, Washington, DC, USA.

2. American Society for Engineering Education, Washington, DC, USA.

3. Center for Nanoscale Science and Technology, National Institutes of Standards and Technology, Gaithersburg, MD, USA.

4. I. Institute of Physics (IA), RWTH Aachen University, Aachen, Germany.

*chase.ellis@nrl.navy.mil

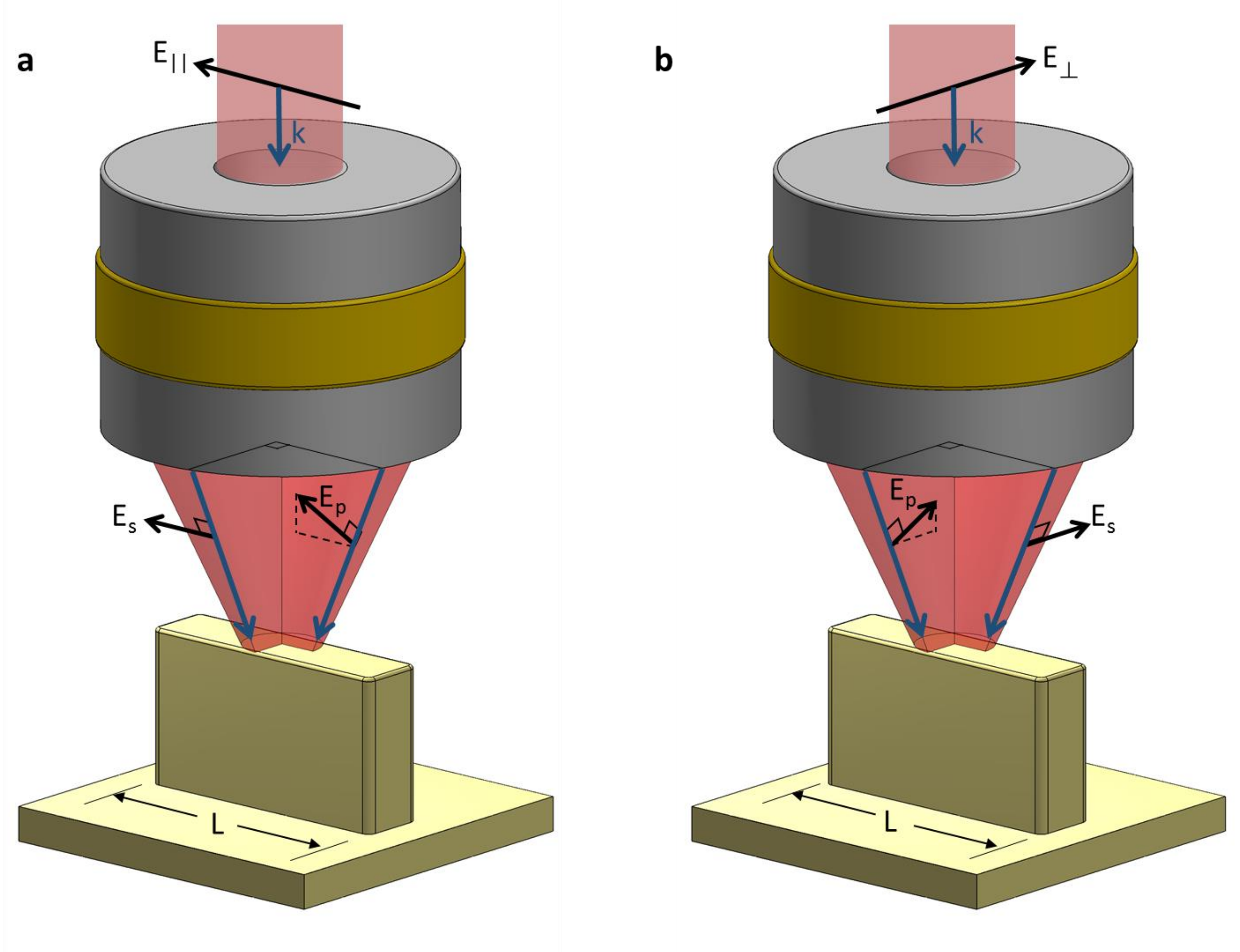

Figure S1: Illustration of sample illumination. The sample is illuminated by a 0.4 NA, all-reflective, reverse Cassegrain objective. Prior to entering the objective, light is polarized nominally (a) parallel or (b) perpendicular to the length of the pillars (depicted by dimension L in figure). For each polarization, the figure depicts the orientation of the polarization with respect to the pillar for light with a plane of incidence parallel and perpendicular to the pillar length. Due to the conical illumination scheme, both s and p polarized light illuminate the sample simultaneously. As depicted, the s polarization is purely aligned with either the width or length axis of the pillar. In contrast, the p polarization also has a component oriented along the height of the pillar, which enables the excitation of longitudinal surface phonon polariton modes.

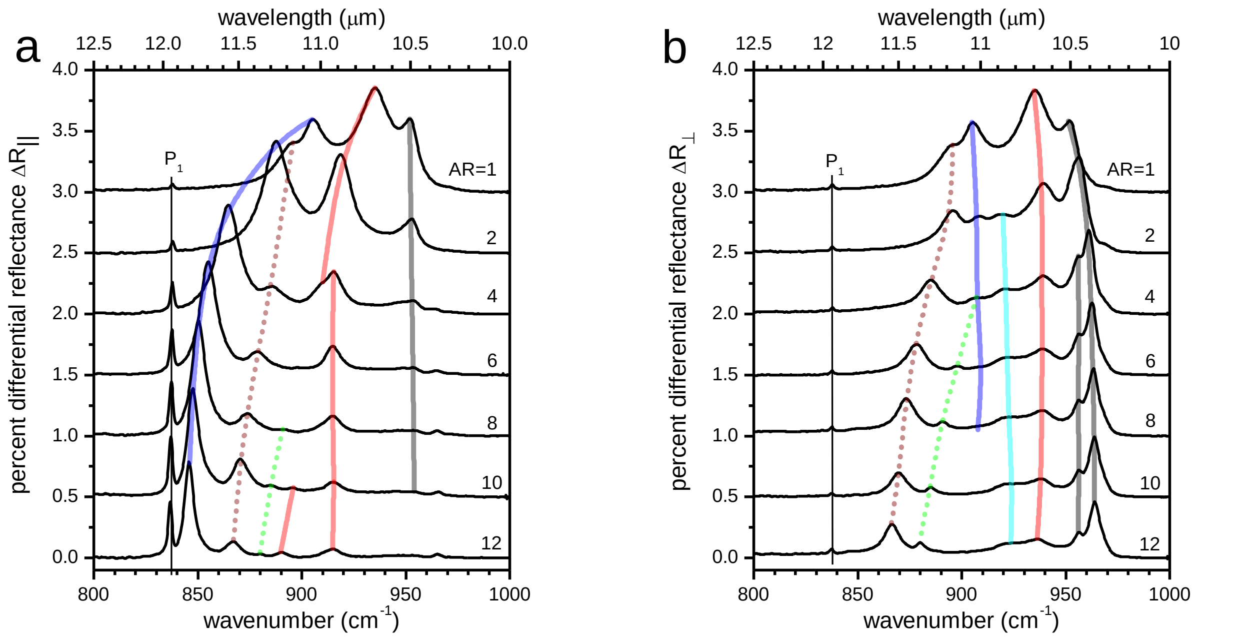

Figure S2: Polarized differential reflectance ($\Delta R_p = R_{\text{SiC}} - R_{\text{SiC \& Pillars}}$) measurements of pillar arrays with aspect ratio varying from 1-12. The incident polarization is oriented (a) parallel and (b) perpendicular to the length of the pillar. Thick solid and dotted guidelines trace the spectral evolution of SPhP resonances with varying aspect ratio for transverse and longitudinal resonances respectively. The guideline colors are consistent with the symbol colors used in Figure 3 of the main text, for easy comparison. In addition, thin, nearly-vertical, guidelines labeled as P_1 indicate the spectral position of the zone-folded LO phonon of 4H-SiC, which is not normally IR active. As shown in panel (a), for small ARs, when all $E_{lwh}^{\|}$ SPhP modes are spectrally separated from P_1, the phonon mode remains inactive. However, as the $E_{100}^{\|}$ SPhP and P_1 phonon mode coalesce at higher ARs, the spectral weight of P_1 increases, indicating a clear coupling between SPhPs and zone folded phonons.

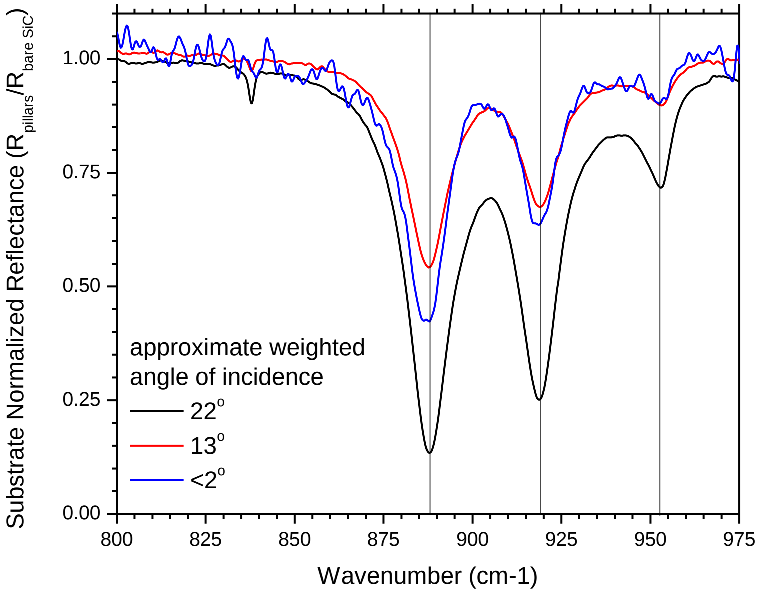

Figure S3: Dependence of SPhP spectra on angle of incidence. Normalized Reflectance Spectra of SPhP resonances with weighted angles of incidence varying from less than 2° to 22° for $AR = 2$ pillar array. In general, the spectral position of resonances is invariant under changes in the angle of incidence.

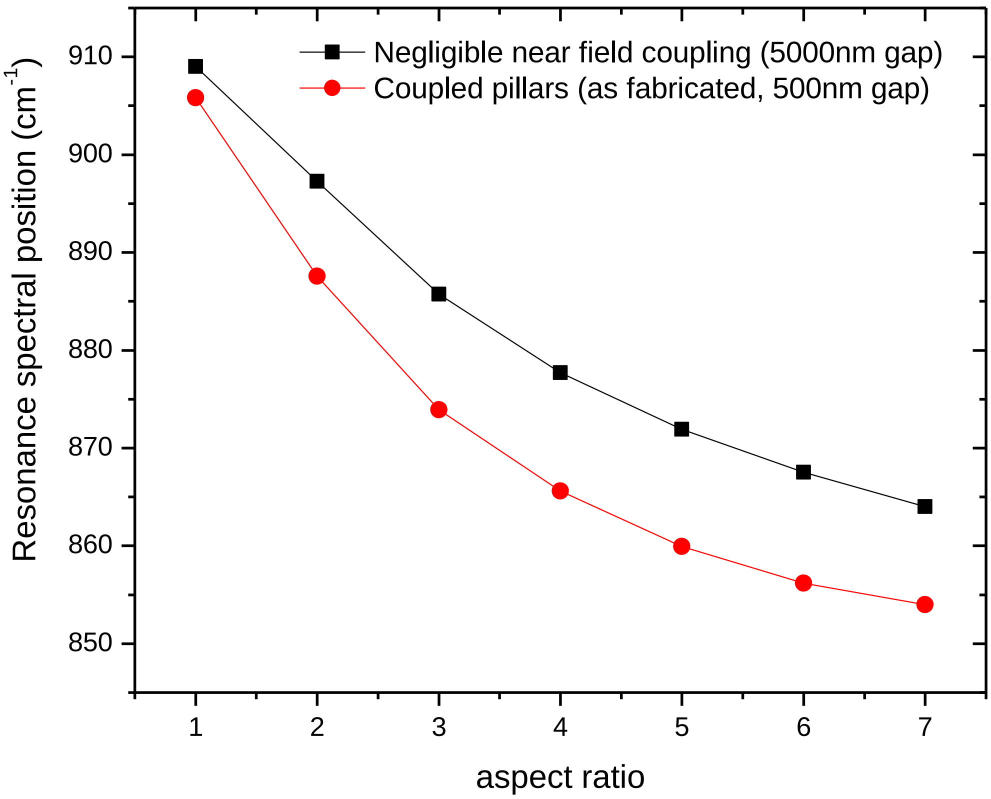

Figure S4: Simulations showing the effect of near field coupling on the spectral position of $E_{100}^{\parallel}$. Black, square symbols show the results of simulations $E_{100}^{\parallel}$ resonance positions when coupling is reduced by separating neighboring pillars by a 5000 nm gap. Red, circle symbols show the results of simulations calculating the spectral position of the same resonance, but with a 500 nm gap separating the pillars, which results in significant near field coupling between adjacent pillars and an overall redshift of the resonance. As shown, as the aspect ratio increases the redshift due to coupling also increases.

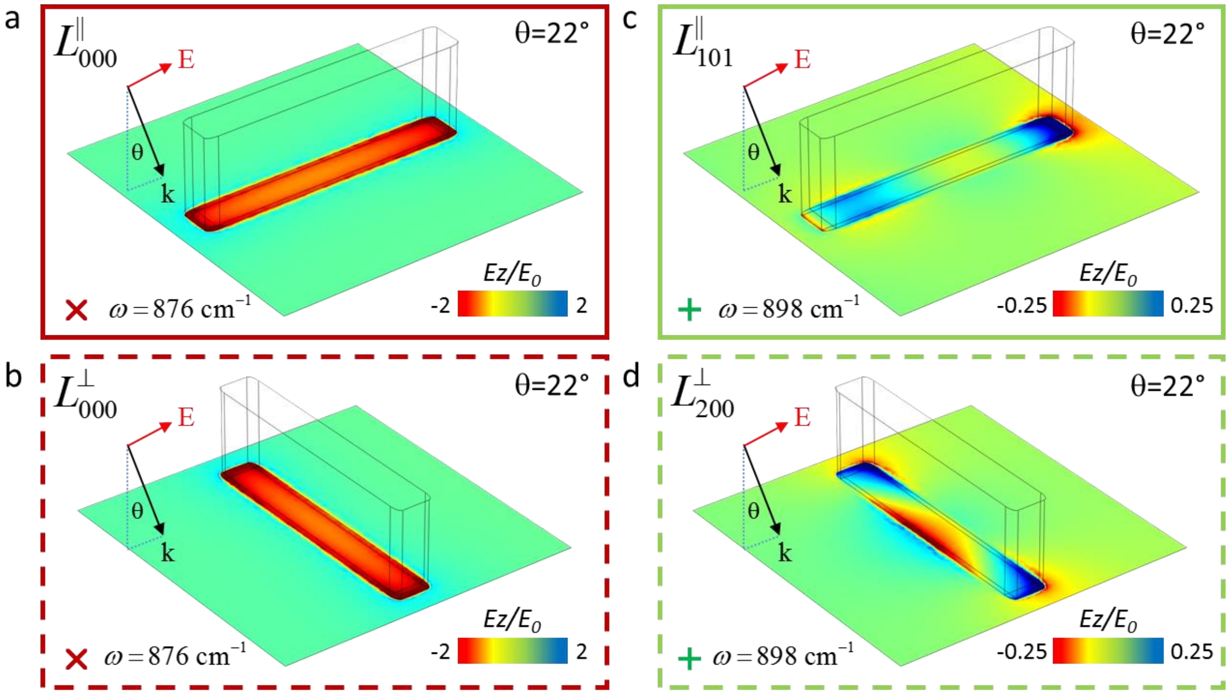

Figure S5: Simulations of the E_z electric field component (oriented along the height of the pillar) in the plane 5 nm below the $AR = 7$ pillar base for the longitudinal modes: (a-b) monopole (L_{000}^{p}) and (c-d) $L_{101}^{\parallel}$ and $L_{200}^{\perp}$. All fields shown are normalized to the incoming field E_0. Panels (a) and (b) show that the E_z / E_0 electric field profile is similar for the spectrally degenerate monopole mode that is excited by the parallel and perpendicular polarized light, respectively. This is in contrast to the spectrally-degenerate, higher-order longitudinal modes shown in panels (c) and (d), which reveal significantly different modal profiles for the parallel ($L_{101}^{\parallel}$) and perpendicular ($L_{200}^{\perp}$) polarizations.
